# Healthcare Consultations for People with Chronic Conditions and Disabilities: Managing Cyber-Victimisation Impact and Training Needs

**DOI:** 10.1177/11786329251386909

**Published:** 2025-10-31

**Authors:** Zhraa Alhaboby, Lorna Rouse, Robin Hadley, Elango Vijaykumar, Haider Al-Khateeb

**Affiliations:** 1 School of Life, Health & Chemical Sciences, The Open University, Milton Keynes, UK; 2 Department of Life Sciences, Manchester Metropolitan University, UK; 3 National Research Lead for Modality Partnership, Horley, UK; 4 Cyber Security Innovation (CSI) Research Centre, Department of Business Analytics and Information Systems, Aston University, Birmingham, UK

**Keywords:** long-term conditions, impairments, patient support strategies, health communication, professional development in healthcare, digital safeguarding

## Abstract

**Background:**

Cyber-victimisation is a growing public health challenge, particularly for people with long-term conditions and disabilities. These individuals face complex challenges in managing health, compounded by experiences of discrimination and insufficient access to appropriate support.

**Aim:**

This study examines healthcare professionals’ encounters with patients who have long-term conditions or disabilities and reported cyber-victimisation. It focuses on the scope of these experiences in healthcare, impact on patients, healthcare professionals’ awareness, and perceived training needs.

**Method:**

A mixed-methods survey was conducted with UK-based healthcare professionals, recruited through the Modality Super GP partnership, social media, and contacting relevant organisations.

**Results:**

The participant sample comprised 118 healthcare professionals, with a mean of 20.72 years of professional experience (SD = 13.72). Among them, 33.90% encountered patients affected by cyber-victimisation, and of these, 82.50% indicated that such experiences had a detrimental impact on their patients’ health. Reported impacts were on mental health, social relationships, lifestyle, physical complications, missing routine appointments, changes to medications, and lab tests. Qualitative themes included mental health consequences, worsening of chronic conditions, increased vulnerability due to certain conditions, trust and stigma, and varied professional awareness. Among those asked about training (n = 77), 58.44% supported research-informed programmes, with preferred formats being interactive media, workshops, and printed materials.

**Conclusion:**

Findings confirm that cyber-victimisation of this group is prevalent in healthcare, yet support and awareness remain limited. Training is needed to equip professionals to assist affected patients. Future research should explore interdisciplinary strategies to strengthen healthcare responses and embed cyber-victimisation awareness into public health policy.

## Key Messages

- Cyber-victimisation is a public health concern that affects people with long-term conditions or disabilities and is increasingly encountered in healthcare settings.
- Healthcare professionals play a crucial role in supporting patients affected by cyber-victimisation by providing both medical care and essential referrals. However, gaps in understanding and training hinder their efforts to offer effective support.
- Training is needed to support recognition, communication, and response to the health impact of cyber-victimisation on people with long-term conditions and disabilites.
- The issue is complex, and requires multidisciplinary work to increase awareness on cyber-victimisation impacts, inform practice, and influence the wider public health policy.

## Introduction

Healthcare professionals (HCPs) have a well-known role in supporting victims of violence.^1^ Research both internationally and in the United Kingdom (UK) has highlighted the involvement of HCPs in supporting patients affected by various forms of traditional victimisation, such as school bullying, workplace bullying, stalking, and domestic abuse.^1-4^

In the UK’s healthcare system, general practitioners (GPs) are the initial point of contact for patients and act as gatekeepers to the wider healthcare system. They are responsible for referrals to specialists and play a central role in the routine follow-up and management of people with long-term conditions. Alongside GPs, a range of healthcare professionals such as nurses, consultants, psychologists, and allied health practitioners are involved in providing care, each with varying levels of patient interaction depending on their role and clinical setting.^5,6^ It is documented that GPs in the UK play a vital role in identifying and supporting young people who have experienced bullying, including physical and psychological abuse.^2^ Similarly, both GPs and school nurses frequently support young people who disclose experiences of bullying, both offline and online.^4,7,8^ Their role includes recognising cases of victimisation and helping to manage its physiological and psychological health impacts.^1,4^

With the increasing use of electronic communication, cyber-victimisation has emerged as a potentially more serious and urgent public health challenge.^9-13^ Although definitions of cyber-victimisation share commonalities with traditional offline forms of victimisation, such as repeated intended harm, cyber-victimisation is distinguished by taking place online or through the use of electronic communication.^11^ Cyber-victimisation takes various forms, including cyber-harassment, cyber-bullying, cyber-stalking, impersonation, and cyber-hate.^14-17^ The terminology used to describe these experiences varies based on factors such as frequency, power imbalance, and underlying motivation. In this research, “cyber-victimisation” is used as an umbrella term to encompass these subcategories.

Cyber-victimisation is a particularly prevalent issue concerning people with chronic conditions and disabled people.^18,19^ According to a report of online bullying in England and Wales by the Office for National Statistics, cyber-bullying is more prevalent amongst those with disabilities (28%) than those without disabilities (18%).^18^ Research showed that 45% of adults with long-term conditions in the UK-based sample had experienced online abuse.^10^ The majority of victims (69%) perceived a worsened self-efficacy for managing chronic diseases. Formal support was generally rated poor, with only 24.5% of victims having disclosed this experience to their physicians.^10^

It has been argued that HCPs including primary care practitioners are especially well placed to support patients who have experienced offline and online victimisation through: identifying at risk patients, recognising or screening for bullying in children, signposting education, and resources on managing victimisation and making referrals for additional psychological or psychiatric interventions where appropriate.^1,20^ Further, it is documented that victims of cyber-abuse may disclose experiences of victimisation to their GPs.^2,13,21^ Victims may present to primary care with a range of symptoms linked to their experience, such as headaches and abdominal symptoms, or signs of depression or anxiety.^10,22^ Additionally, stress is a common complaint in primary care^23-25^ and it is associated with cyber-victimisation,^11,17^ often manifesting as psychosomatic symptoms. Hence, GPs working in the UK can be seen as the first point of contact and gatekeepers to the referral system required by the victims. People with chronic conditions in the UK experienced health deterioration due to being victimised online,^26^ and reported unintended changes to follow-up with HCPs such as specialist (6.6%), GPs (15.16%), counselling sessions (15.6%), and other HCPs (6.3%).^10^ Hence, input from HCPs with regular contact with patients is also necessary to understand these encounters. The input from HCPs who were not contacted by the victims is also helpful to understand their view from a biomedical point of view.^27^

From a biomedical perspective, prolonged exposure to cyber-victimisation is common,^10^ and can lead to sustained stress responses, triggering neurohormonal changes that may directly affect physical health.^27,28^ Additionally, stress may influence patients’ adherence to treatment plans, dietary choices, and self-management behaviours. Thus, it is crucial to explore whether healthcare professionals recognise the health implications of cyber-victimisation through a biomedical lens.

The assumed role of HCPs in the self-management of chronic diseases is to explain to patients and enable effective self-management of the condition.^29^ This is challenging in the case of cyber-victimisation because HCPs may recognise the problem, but do not provide effective support.^10,30^ This is complicated by victim’s perceptions of not being taken seriously by different agencies.^31^ The academic literature on cyber-victimisation has called for greater involvement and training of HCPs in recognising and supporting young victims.^1,2,8,32^ Nevertheless, understanding of healthcare professionals’ perceptions and experiences relating to the cyber-victimisation of their patients, including adults, remains limited. A possible challenge in research is the limited participation by HCPs in the UK due to recognised heavy workload and short consultation time.^33^

This study aimed to examine healthcare professionals’ encounters with patients with long-term condition or disabilities who complained of cyber-victimisation. The objectives were to scope these experiences, understand perceived health impacts, examine awareness/misconceptions among healthcare professionals, and whether there is a perceived need for training or guidance in this area to improve practice. It deployed a mixed-method approach to scope the experiences using quantitative data, and further explore meanings using the qualitative data.

## Methodology

A mixed-methods survey design was employed to explore healthcare professionals’ experiences, particularly given the limited existing literature on this topic. Surveys have proven a valuable tool for gathering the views of HCPs on a range of topics^33,34^ and for investigating experiences relating to cyber-victimisation.^2,31^ A mixed-methods approach is especially useful in new or unexplored topics because it allows the inclusion of views and experiences which go beyond multiple-choice options.^31^ By combining quantitative and qualitative methods, the study benefits from the strengths of both; quantitative data helps scope these experiences, while qualitative data provides depth and context. The integration of findings enables a more nuanced interpretation, allowing each component to inform and enhance the other, which is an approach especially valuable in under-researched or sensitive areas such as cyber-victimisation in healthcare settings. In this study, priority was given to the qualitative component due to the scarcity of in-depth research on cyber-victimisation reporting in healthcare settings.

### Terminology Use

In this study, the terms long-term conditions, chronic conditions, and chronic diseases are used interchangeably, reflecting how they are understood and applied across different disciplines and healthcare settings.

The term *chronic* originates from the Greek word *khronos*, meaning time, and is medically used to describe illnesses that are persistent, recurrent, or slowly progressive over time.^35^ Public health and international health organisations typically reserve the term chronic disease for non-communicable conditions such as cardiovascular disease, cancer, diabetes, and chronic respiratory disorders, which require ongoing management and often persist for a year or more.^36,37^ These are among the leading contributors to global morbidity and mortality.^38^ The public health framing of chronic disease is the one adopted in this research. Furthermore, there is a significant overlap between chronic conditions and disabilities. Many individuals living with chronic illness experience long-term impairments that meet the legal and functional definitions of disability, and vice versa.^39,40^ In the UK context, the Equality Act 2010 defines disability in a way that includes many chronic health conditions due to their long-term impact on daily functioning. Therefore, using these terms interchangeably in this study reflects their overlapping realities and helps capture the full spectrum of healthcare professionals’ encounters with affected patients.

Disability is often stereotyped and its health impacts can be overlooked in clinical practice which is why the questions about impact and biomedical relevance were more focussed on chronic disease or long-term condition terminology to steer respondents from social aspects to health aspects.

### Theoretical Underpinning

The theoretical framework underpinning this study is based on the concept of biographical disruption, which describes chronic conditions as disruptive events that alter every aspect of a person’s life.^41,42^ The diagnosis of a long-term condition may lead individuals to rethink their identity, mobilise psychological and social resources, and adapt to a new reality. This process influences how people manage their symptoms, treatment, lifestyle changes, and social relationships. In this context, cyber-victimisation may act as a second disruption. For people already living with a long-term condition, being targeted online can further undermine their emotional wellbeing, trust in healthcare, and ability to manage their health. Cyber-victimisation was found to change how people perceive themselves and others, disrupt their relationships, and cause long-lasting psychological harm. It affects the same psychosocial resources needed for coping with a chronic illness.^26^ This framework guided the research and provided a foundation to explore healthcare professionals’ awareness, experiences, and views on managing such cases in clinical practice.

### Survey Design

The survey aimed to scope encounters with people who have experienced cyber-victimisation while living with a long-term condition. The survey design was informed by discussions with stakeholders and the literature on cyber-victimisation of people with long-term conditions.^10,11^ It was piloted in a 1-1 discussion with Modality GP partnership, in which the main feedback was to keep the questions short and targeted to suit the audience and time constraints. This was followed by piloting with 3 HCPs to test the functionality, clarity, usability of the online platform, and to obtain input from on the wording or other areas of concern. The question on religion was removed at the piloting stage.

The survey consisted of both closed and open-ended questions. Participants were first asked about their professional background, including their role, years of experience, and area of practice. They were then presented with a brief definition of cyber-victimisation and asked whether they had encountered patients with long-term conditions or disabilities who had experienced such harm. A definition of cyber-victimisation was included within the survey to ensure a consistent understanding among participants. This approach was informed by inconsistencies in terminology identified in the literature^11^ and helped to reduce variability in interpretation.

Subsequent questions explored the perceived impact of cyber-victimisation on patients’ health and well-being, using a multiple choice question which, followed by an open ended question to ask participants about the potential impact from their own clinical experience and medical knowledge. Finally, healthcare professionals were asked whether they would benefit from training on the topic, and what formats they would prefer.

### Ethics

The research received a favourable opinion from the Open University Human Research Ethics Committee (HREC), and no data collection commenced prior to this approval (ref: HREC/4741).

A verification page displayed inclusion criteria and screening questions to verify the participant’s eligibility criteria as recommended by the Ethics Guidelines for Internet-mediated research.^43^ Once the criteria were verified, the potential participants viewed the research information and provided written consent before proceeding to the survey. Responses were anonymised and downloaded collectively for further analysis.

### Recruitment and Data Collection

While existing literature and previous research have often positioned GPs as central gatekeepers to care for individuals affected by forms of violence, the inclusion criteria in this study led to a more diverse representation of healthcare professionals. This provided broader insight into how cyber-victimisation is encountered and addressed across different roles in the healthcare system. The inclusion criteria were: HCPs (trained professionals to provide medical services or treatment to patients), from any gender and ethnic background who currently or previously had clinical experience in the UK, with direct contact with patients living with long-term conditions, based in any healthcare setting, with or without experience with victims of cyber-abuse, regardless of the patient’s age. These include GPs, specialists, consultants, nurses, clinical psychologists, and other HCPs with direct regular consultations with people with long-term conditions in the UK. Exclusion criteria were HCPs in training, HCPs based outside the UK with no prior clinical work in the UK, and those with no direct consultations with patients who have long-term conditions or disabilities.

To overcome HCP workload challenge in the UK, an online design was employed to allow flexibility, and the recruitment included multiple ways of communication and gatekeepers.^34^ Participant recruitment was undertaken via 3 routes; a recruitment call by the project collaborator Modality, a targeted social media campaign and a desktop online search.

Modality is a super GP partnership in the UK with nationwide clinics. The survey was promoted via Modality’s newsletter and internal mailing list. Recruitment also included a campaign on X (formerly Twitter) with 2 tweets collectively receiving 81 likes, 86 retweets, and 2777 link clicks (0.15% engagement rate). While the majority of participants recruited through our collaborator Modality Partnership were GPs, the wider social media campaign helped secure a more diverse sample of healthcare professionals across various disciplines.

The desktop online recruitment strategy was to identify potential participants via non-governmental organisations. The search considered the following criteria: (a) established support groups or organisation, (b) based in the UK or with significant audience from the UK, (c) having terms and policies in their websites aligning with ethics to protect participants, (d) having direct contact with HCPs, and (e) provided contact details for research. Organisations that fit the study criteria were contacted by personalised proforma email with non-respondents sent a follow-up email 7 days later. The email outlined the background to the study, ethical policy, and a link to the online survey. Organisations that were interested helped in “re-tweeting” the survey link.

The sample size was guided by feasibility, including project timeframe and available resources, and took into account the challenges of engaging healthcare professionals.

### Data Collection and Analysis

Respondents accessed the survey via a link to the Qualtrics XM platform. The survey was open for responses between 15th June 2023 and 23rd October 2023. Responses were anonymised and downloaded collectively for further analysis. The quantitative data primarily served to scope the experience, while the qualitative input, which generated rich data and was prioritised in the analysis and reporting, provided additional meaning and depth. The integration of the quantitative and qualitative findings was conducted during the discussion phase by 3 researchers (ZA, LR, and HK), this approach allowed the qualitative themes to provide context and explanation for the survey data, and vice versa. Conducting integration at this level is particularly useful in exploratory research, where emergent insights benefit from the strengths of both methods.

#### Quantitative Data

A total of 180 potential participants checked their eligibility, and 118 respondents were eligible to participate and completed the survey. Respondents had the option to skip questions, which is common in survey-based research, and not all participants are expected to answer every question.^44^ In quantitative analysis, the number of respondents is presented alongside each question. Descriptive statistics were used for quantitative data and were integrated with the qualitative data in the discussion.

#### Qualitative Data and Analysis

The qualitative data were collated from responses to open-ended questions. A total of 114 participants provided free-text responses. Of these, 25 were excluded due to lack of usable content (eg, “n/a,” “no,” “.”) or incomprehensibility. The remaining 89 responses were included in the analysis. Thirty-nine of these responses (44%) exceeded one line in length. In line with good practice for analysing qualitative comments in surveys, the proportion of longer responses is reported above.^45^ Despite the brevity of some responses, consistent patterns and concepts emerged across diverse professional roles. These were systematically analysed to develop coherent and meaningful themes. Each participant was given a code, and the included responses were cross-checked with these codes.

A deductive thematic analysis was applied, so that analysis was driven by the data rather than existing theory. Thematic analysis is a flexible approach which can be applied to a range of textual data including survey responses^46,47^ and has been successfully used in previous analyses of free-text survey data collected from HCPs.^48,49^ We did not exclude comments from participants without direct encounters, they were included to explore general professional understanding, identify perceptions or detect misconceptions, and inform training needs.

Data were entered into the qualitative analysis software NVivo One to organise and support checking the fit of data to themes. Free text responses were read for familiarisation. Responses were read with the research question in mind and initial codes were generated. As advised Braun et al,^47^ responses were considered as an entire data set and themes were created to fit the research question. To ensure analytical rigour, 2 researchers (ZA & LR) discussed and agreed on initial codes. Responses were assigned to these agreed themes and these were reviewed for goodness of fit with the data and combined or revised where necessary into themes and sub-themes.

## Results and Discussion

### Quantitative Results

#### Sample Demographics

The sample included 118 respondents, with 55.08% being female (65/118), and 92.24% having the same gender as the sex assigned at birth (107/116). Respondents ranged in age from 25 to 83 years with a mean age of 46.05 years (SD = 13.61), and 19.49% (23/118) reported having a disability. The sample was predominantly 79.66% (94/118) from a White ethnic background, with representations from Asian, Black, mixed, Arab, and other backgrounds. Participants had an average of 20.72 (SD = 13.72) years of experience in healthcare, with 83.05% currently practising. The most common areas of practice were nursing (33.90%), general practice (14.41%), and specialist roles (11.86%), in addition to other areas. Table 1 summarises the sample’s demographics.

**Table 1.** Respondent Responses to Demographic Questions.

Characteristic	N (%)
Sex, N = 118	
Female	65 (55.08)
Male	46 (38.98)
Not specified	7 (5.93)
Ethic background, N = 118	
White	94 (79.66)
Mixed/multiple	3 (2.54)
Asian/Asian British	6 (5.08)
Black	3 (2.54)
Arab	2 (1.69)
Other	4 (3.39)
Not specified	6 (5.08)
Disability, N = 118	
Yes	23 (19.49)
No	91 (77.12)
Not specified	4 (3.39)
Currently practising as a healthcare professional, N = 118	
Yes	98 (83.05)
No	17 14.41)
Not specified	3 (2.54)

The most common areas of practice were nursing (33.90%), general practice (14.41%), and specialist roles (11.86%), in addition to other areas. Beyond predefined categories, participants could specify their specialty under “Other.” Several specialities appeared frequently enough (2 or more mentions) to warrant separate categorisation, these have been separated from the general “Other” category and incorporated into the main table for clarity and representation. Table 2 summarises the diversity of the sample’s areas of speciality.

**Table 2.** Respondents’ Specified Areas of Specialty or Practice.

Area of practice/specialty, N = 118	N (%)
Nurse, please specify:	40 (33.90)
	Specialities in free text box: general, critical care, mental health, community, primary care, specialist roles (e.g., cardiology, respiratory, endocrine), education, and research.
General practitioner (GP)	17 (14.41)
Specialists (eg, cardiologists, pulmonologists, neurologists), please specify:	14 (11.86)
	Specialities in free text box: cardiology, gynaecology, orthopaedics, anaesthesia, oncology, haematology, geriatrics, paediatric neurology, and neurology.
Psychiatrists	6 (5.08)
Clinical psychologist	4 (3.39)
Behavioural psychologist or psychotherapist	4 (3.39)
Physiotherapist	4 (3.39)
Optometrist	2 (1.69)
Pharmacist	2 (1.69)
Healthcare assistant	2 (1.69)
Paramedic	2 (1.69)
Occupational therapist	2 (1.69)
Dental/Dentist	2 (1.69)
Radiographer	2 (1.69)
Operating department practitioner	2 (1.69)
Speech and language therapist	2 (1.69)
Ophthalmologist	2 (1.69)
Other, please specify	9 (7.63)
	Areas in this category: Dietitians or nutritionist, respiratory therapist, phlebotomist, biomedical scientist, training community worker, psychosexual therapist, foot health, restrictive practice lead, physician associate.

Additional exploratory analyses were conducted to examine whether the likelihood of encountering patients who had experienced cyber-victimisation varied by healthcare professionals’ demographic or professional characteristics. No statistically significant association was found between years of experience and cyber-victimisation disclosures (χ² = 3.98, *P* = .86), nor between participants’ sex and such disclosures (χ² = 0.74, *P* = .69). Similarly, no significant difference emerged across the 3 most represented professional groups who were nurses, GPs, and specialist (χ² = 4.20, *P* = .12).

#### Views and Experiences on the Impact of Cyber-Victimisation

The participants were given the definition of cyber-victimisation in this study and then asked whether they had patients who complained of cyber-victimisation, 33.90% (40/118) of HCPs said “yes,” and of those 82.50% (33/40) said that the patient had reported the impact of this experience on their health. The most frequently reported areas of impact are reported in Table 3. Notably, 16 participants selected more than one impact, indicating that cyber-victimisation may have multifaceted effects on patient wellbeing.

**Table 3.** Responses to Multiple Choice Questions About Views and Experiences on the Impact of Cyber-victimisation.

Question	N (%)
Have you ever encountered a patient with a chronic disease or disability who was complaining of such negative online experiences? N = 118	
Yes	40 (33.90)
Not sure	17 (14.41)
No	61 (51.69)
Did the patient report the impact of this experience on their health? N = 40	
Yes	33 (82.50)
Not sure	7 (17.50)
No	0
What was the impact? N = 58	
Mental health	22 (37.93)
Physical health	6 (10.34)
Social impact	13 (22.41)
Lab test	1 (1.72)
Change to medications	3 (5.17)
Change to lifestyle	7 (12.07)
Impact on routine appointments	5 (8.62)
Other, provide examples	1 (1.72) Free text box: They became less trusting in others.

Another notable finding is that 17 respondents (14.05%) indicated that they were unsure whether they had encountered patients affected by cyber-victimisation, suggesting a degree of ambiguity or uncertainty in recognising such experiences in clinical practice.

#### Views on the Role of Training to Raise Awareness of Cyber-Victimisation

Participants were asked whether they thought that HCPs would benefit from research-informed training to raise awareness on cyber-victimisation impact on people with chronic conditions. Of the 77 respondents to this question, 58.44% (45/77) replied yes. Participants were also asked about their preferred format and whether they would be interested in taking this training or recommending it to colleagues, the answers are summarised in Table 4.

**Table 4.** Responses to Multiple Choice Questions on the Role of Training in Raising Awareness of Cyber-victimisation.

Question	N (%)
Do you think healthcare professionals would benefit from research-informed training to raise awareness on cyber-victimisation impact on people with chronic conditions? N = 77	
Yes	45 (58.44)
Not sure	18 (23.38)
No	14 (18.18)
What would be your preferred format of training? N = 86* *participants were able to select more than one preferred method of training.	
Workshop	26 (30.23)
Interactive media	38 (44.19)
Booklet	6 (6.98)
Leaflet or poster	13 (15.12)
Other, such as	3 (3.49)
In-person	1
Webinar	2
Will you be interested to take this training or recommend it to colleagues? N = 63	
Yes	32 (50.79)
Maybe	28 (44.44)
No	3 (4.76)

### Qualitative Findings

Thematic analysis identified 7 key themes: (1) Psychological and Psychiatric Impact, (2) Impact on Chronic Disease Self-Management, (3) Relationship with Healthcare Providers, (4) Social Impact and Isolation, (5) Patients’ Conditions and Individual Characteristics, (6) Responsibility to Intervene, and (7) Unrecognised or Uncertain Impact. Some of these themes included sub-themes as detailed below.

#### Theme 1: Psychological and Psychiatric Impact

Many participants described the serious consequences of cyber-victimisation on mental health, highlighting both psychological and psychiatric impacts. Two subthemes emerged from the responses.

The first subtheme, ***Specific impact on mental health and wellbeing***, focuses on how HCPs defined the negative effects of cyber-victimisation. They reported that patients may experience feelings of isolation, embarrassment, fear, distress, and vulnerability. Additionally, HCPs expressed concerns that negative online experiences could exacerbate symptoms in patients with pre-existing mental health conditions. They highlighted specific mental health conditions or complications that may be triggered or worsened by cyber-victimisation, including depression, anxiety, insomnia, low self-esteem, self-harm, and suicidality. For example, a participant stated:

Further increases fear and isolation both present due to chronic health conditions, reduces the size of their “world”. Increase rates of mental health disturbances.

(J14, white female, psychiatrist, encountered patients with cyber-victimisation experience)

Negatively affects their outlook of themselves, lowers self esteem, worse outcomes on depression and anxiety scales.

(J01, black female, psychiatrist, have not encountered patients with cyber-victimisation experience)

Concerned re the use of diagnosis by tiktok/overuse of similar platforms by usually young adult females who have ?psychiatric illness and self harming behaviour, and use social media/vlogging as an outlet . . . Have dealt with patients who have attempted suicide secondary to social media treatment.

(Jn20, white female, speech and language therapist, encountered patients with cyber-victimisation experience)

The second subtheme was the ***Worsening of existing chronic conditions***. Several responses indicated that for patients with chronic conditions, there was worsening in existing chronic conditions as a consequence to mental health impact. HCPs describe the impact on physical symptoms either directly such as *“flare ups in pain”* or indirectly by influencing self-management behaviours through discouraging participation in treatments or engaging in unhealthy behaviours such as poor diet. For example, respondents described the impact of cyber-victimisation as:

Worsening mental health which can then lead to poor outcomes for their physical health.

(J06, white male, GP, encountered patients with CV experience)

HCPs further reported that since living with a long-term condition can impact negatively on a person’s mental wellbeing, cyber-victimisation may be especially difficult for this group. It may exacerbate the ongoing emotional impact of living with long-term conditions such as feelings of isolation and experience of depression or anxiety.

#### Theme 2: Impact on Chronic Disease Self-Management

HCPs described cyber-victimisation as impacting on elements of self-management of the person’s chronic condition. Aspects of self-management mentioned included engaging in unhealthy behaviours such as poor diet and changes in medication. Some participants described how the impact on mental health as described in theme 1 would impact self-management.

Deterioration of mental health which can have an impact on patients coping skills who will then use excess alcohol and/or drugs to self medicate.

(Jn23b, white female, addiction nurse, encountered patients with cyber-victimisation experience)

HCPs also proposed that negative online experiences may impact self-management by leading to a withdrawal of participation in treatments, support and activities of daily living. Specifically, patients may become reluctant to engage with healthcare and to leave their homes due to feelings of isolation and stigma.

impact on confidence leaving the house, socialising, accessing services - in worse case scenarios (e.g. doing, credible threats) impact on physical safety,need to temporarily relocate, impact on access to support/normal aids & equipment.

(J14, white, preferred not to share sex, physiotherapist, not sure if encountered patients with cyber-victimisation experience)

#### Theme 3: Relationship with Healthcare Providers

Many participants described the negative impact of cyber-victimisation on a patient’s relationships with their healthcare provider. The most frequently mentioned impact was that the experience of cyber-victimisation may discourage patients with chronic conditions from seeking or accepting help and treatment. Several respondents explained that reluctance to access support was due to cyber-victimisation making them feel shame (see theme on stigma below) or that they are “responsible” for their condition and thus unentitled to or undeserving of help.

May make them feel vulnerable / 2nd class citizen / be subjected to misinformation / discourage them from taking offered treatments or support

(J11, white, female, physiotherapist, not sure if encountered patients with cyber-victimisation experience)

People with multiple and/or chronic conditions can often feel that social media infers that they are solely responsible for their own health conditions.

(Participant J11b, white, female, nurse specialist, not encountered patients with cyber-victimisation experience)

Within this theme, a few responses described how cyber-victimisation may impact a person’s relationship with their healthcare providers by leading them to believe that HCPs should be mistrusted. Online interactions may tell them that they have been *“subjected to misinformation”* or lead them to *“expect a bad experience.”* One response gives an example of a patient being told online that they are *“being lied to,”* when referring to information given during a hospital stay.

#### Theme 4: Social Impact and Isolation

This was a major theme in the dataset and it covers how cyber-victimisation leads to social isolation. Many responses referred to a general isolation or feeling isolated whilst several others described a specific social impact which can be broadly divided into 3 subthemes.

The first subtheme is the **Withdrawal from online sources of support**. The respondents described leaving online sources of support because the internet becomes a *“threatening”* space. In line with the first theme above, respondents described a double impact for those with chronic illness. Withdrawal from online support may be especially difficult for people who live with chronic illness since this group is more likely to experience social isolation in terms of real-world interactions and are thus perceived to be more engaged in interactions with online communities for social support and *“connection.”*

Online resources can be a vital part of connection and alleviation from social isolation. If these spaces become negatively associated or begin to produce negative outcomes for the patient this can influence quality of life, including suicidality drastically. . .

(J05, white, female, psychosexual therapist, has not encountered patients with cyber-victimisation experience)

The internet is often a place where people with chronic and long-term conditions can come together to find support. If their experience of the internet becomes dangerous and/or threatening, not only is a valuable source of support lost, but an extra trauma is added to the trauma of having a long-term condition.

(J01b, white female, clinical psychologist, encountered patients with cyber-victimisation experience)

The second subtheme is ***Withdrawal from real-world interactions***, which was highlighted how social isolation resulting from cyber-victimisation might also involve withdrawal from real-world interactions due to fear or lowered confidence and self-esteem.

People with multiple and/or chronic conditions can often feel that social media infers that they are solely responsible for their own health conditions. They can often not want to leave their own homes due to discrimination.

(J11a, white, female, nurse specialist, have not encountered patients with cyber-victimisation experience)

The third subtheme was the ***Experience of stigma*** which highlighted that patients often experience shame and stigma due to negative online comments, making them feel different from others. Several HCPs described patients as feeling “ostracised,” “isolated,” and “abnormal,” with some being perceived as “faking” their illness or as “scroungers.” Beyond leading to social isolation, stigma also affects self-management and relationships with healthcare providers by discouraging help-seeking behaviours, as previously discussed.

Causes difficulty seeking help Makes them embarrassed to describe symptoms (e.g. they can’t describe their condition as “triggered” by something as this has become an Internet joke) Theirs posts are shared on “fake illness” pages of sites like reddit and 4chan, and then they and their social media are hounded by people calling them fake, scroungers, attention seekers etc, until they withdraw from interacting with others.

(J01c, white, female, nutritionist, encountered patients with cyber-victimisation experience)

People with specific characteristics or conditions were described as more likely to be stigmatised online due to the wider socio-political context (see also theme 5).

#### Theme 5: Patients’ Conditions and Individual Characteristics

Many respondents reported that whether cyber-victimisation has an impact, and the extent of that impact is dependent on patient characteristics and/or their chronic conditions. This feeds into the wider theme that the impact of negative online experiences is variable. Where respondents gave a further explanation, the characteristics or conditions were described as making the person less or more of a target for cyber-victimisation and influencing the importance of the experience to the person. This theme was divided into 2 subthemes.

The first subtheme is the ***type of conditions***. Many HCPs expressed the view that having a long-term condition may make the impact of negative online experiences especially difficult because it aggravates an already difficult experience. Having a specific type of chronic condition (mental health, long-term COVID) was described as making the person particularly at risk to the negative impact of cyber-victimisation.

People with Long Covid are particularly vulnerable to bullying and/or harassment, as unfortunately, there are people within our society who, for their own political reasons, do no not believe the condition exists.

(J01b, white female, clinical psychologist, encountered patients with cyber-victimisation experience)

The second subtheme is ***Demographic and social factors*.** This subtheme refers to how some factors, individual and social, were described by HCPs as potentially increasing or limiting a patient’s risk of the negative impact of cyber-victimisation. The characteristics referenced were: age (school children, younger people/teenagers, older people), gender, and neurodivergent.

I see mainly children. Social media use by young people is much more prolific than in adults. It allows the playground bullies into the child’s home and bedroom. There is no respite from the misery of school bullying. It causes depression, anxiety, suicidal ideation and poor self confidence / hatred.

(J05c, white male, paediatric neurologist, has encountered patients with cyber-victimisation experience)

Interestingly, older age was referred to as a characteristic which may make the individual less susceptible to cyber-victimisation, due to limited internet access as above, and a characteristic which increases the risk of being targeted.

From experience, I am aware the elder generation who are generally over 80 years of age are more likely to be targeted for victimisation and/or online fraud and may benefit from some trusted literature to keep them safe online.

(A18, White male, specialist, encountered patients with cyber-victimisation experience)

Within this theme, a few responses report that the groups they work with have limited risk of impact from negative online experiences because their age or condition means that they have limited online access/experience (older people, young children, and people with severe learning disability), for example:

I’ve never known such incidents before. It might be because the patients I treat are too old to use social media or Internet overall.

(Jn24d, Arab, male, specialist/geriatrics, have not encountered patients with cyber-victimisation experience)

It is noted a few of the responses which suggest characteristics that limit vulnerability were made followed a statement that the healthcare professional had not heard of their patients experiencing cyber-victimisation and limited internet access for the group of patients they work with as a possible explanation for this (see theme 7).

#### Theme 6: Responsibility to Intervene

In this theme, many HCPs expressed views on whether and how the impact of cyber-victimisation might be ameliorated, including comments on the role that HCPs might play in this.

The impact of cyber-victimisation was viewed as a problem that requires some kind of intervention, including responses which appear to give HCPs a role in or responsibility for providing support.

If a patient expresses concern, the health care professional dealing with them should help them address issues.

(Jn17, white female, primary care nurse, has not encountered patients with cyber-victimisation experience)

Some HCPs also stated that providing support or interventions can be difficult because HCPs lack training or awareness of cyber-victimisation, or have limited time to tackle this issue.

I think it’s something that has become widespread quite quickly and professionals have not been trained to spot patients who might have been victimised, or know how to support them

( J09, white, female, hepatologist, encountered patients with cyber-victimisation experience)

In Theatres, they often blurt out their issues pre anaesthesia. Which for staff is difficult to handle. We only have moments to reassure. Thinking why wasn’t this handed over by ward staff? But, they didn’t know. Anaesthesia is a conundrum. Some simply trust & submit. Some hate the feeling of not being in control. It the latter category who blurt out all their issues, more commonly feelings of loneliness & cyber bullying

(Jn24c, mixed ethnicity, female, Senior Operating Department Practitioner, encountered patients with cyber-victimisation experience)

One response also expressed the view that in order to be effective, any training would need to be carefully designed and does not exhaust busy HCPs with hours of, sometimes irrelevant, training. Two suggestions referred to support or interventions aimed at equipping patients/people with long-term conditions who have experienced cyber-victimisation with skills on how to block or take back control.

#### Theme 7: Unrecognised or Uncertain Impact

The themes set out above all come under the distinct pattern that cyber-victimisation is problematic and harming patients with chronic conditions. However, it is important to acknowledge that in 23 responses HCPs said either that they thought that negative online experiences had no, unknown or limited impact on people living with long-term conditions. Some of these participants also expressed their views using negative stereotypes, including gendered language.

The majority of responses in this theme consisted of brief replies in which respondents stated that they had no knowledge or experience of cyber-victimisation and how it might impact their patients. As discussed above, a few stated that cyber-victimisation may impact groups they do not work with to explain their lack of experience. On the other hand, 2 of the respondents mentioned that while they had wide experience in healthcare for people with chronic conditions they did not identify cyber-victimisation as a problem.

*“All my patients have chronic conditions. None have ever complained of it”*,

(Participant J08, white male, anaesthesia, has not encountered patients with CV experience)

Four responses much more clearly state that they do not believe that cyber-victimisation is a problem for people with chronic conditions and one person stated that they are only aware of positive online experience.

No professional experience. People have only reported positive online experiences in patient groups.

(Participant J21, white, did not specify sex, GP, has not encountered patients with CV experience)

It’s all in the mind and they should all man up.

(J07, male, did not specify ethnicity, did not specify speciality, have not encountered patients with cyber-victimisation experience)

Although responses to this theme are not detailed, they do flag that healthcare professionals’ perceptions of the impact of cyber-victimisation may include the view that it is outside of their experience or that it is not an important or even a real problem for people living with long-term conditions, making interventions and training necessary.

## Discussion

This study investigated experiences with patients with long-term conditions, specifically whether cyber-victimisation was a presenting complaint in a healthcare setting and its potential impact on the patient’s health. The sample consisted of healthcare professionals working in diverse areas of practice in the UK. About one-third of the sample (33.90%) reported encountering patients affected by cyber-victimisation, reinforcing previous UK-based studies that have documented the growing intersection between digital harms and public health.^10,19,26^ The qualitative themes gave further depth to the extent and nature of these encounters, showing the impact on self-management and overall wellbeing. The study emphasises the need for public health interventions, and the importance of equiping HCPs with appropriate training to manage patients and make appropriate referrals.

The most frequently reported impact of cyber-victimisation was on mental health (37.93%), including emotional distress, anxiety, and depression. This was also a prominent theme in the qualitative data. These findings are consistent with existing literature, which has established strong links between cyber-victimisation and adverse mental health outcomes.^17,50,51^ For individuals with long-term conditions, the exacerbation of pre-existing mental health conditions due to cyber-victimisation could further compromise self-management and long-term wellbeing and this was further reflected in the qualitative themes. This aligns with public health research demonstrating how psychological distress can negatively influence chronic conditions’ outcomes.^52^ However, while the link between cyber-victimisation and mental health impact is recognised, as is the association between psychosocial distress and poor health outcomes in chronic conditions, there is no direct or adequate action currently in place at a national level to mitigate it.

Importantly, this study provides a novel framing of cyber-victimisation as not only a mental health concern but a public health issue that disrupts chronic condition management and engages the responsibilities of healthcare professionals. Beyond mental health, cyber-victimisation was reported to affect the self-management plan of the patients which were described in detail in the themes. These findings support previous research indicating that online abuse can lead to non-adherence to treatment plans, changes in diet and exercise routines, and increased feelings of helplessness.^10,26^ The participants shared their perceptions on how these experiences impact self-management, medication adherence, and psychological resilience, which is in line with the theoretical unpinning in this research. The study builds on the concept of biographical disruption and positions cyber-victimisation as an additional disruptive event in the trajectory of long-term conditions.^26,41,42^ This perspective contributes to emerging discussions on the digital determinants of health. Hence, public health professionals and policymakers must be aware of these digital stressors and their implications.

An important finding of this study is the perceived shame and stigma associated with experiencing cyber-victimisation and seen as a reason to delay in seeking healthcare. The literature suggests that victims of online abuse generally face underestimation or are not believed when reporting their experiences, which inadvertently could have lowered trust in public services and HCPs.^31,53^ Moreover, misinformation encountered online can impact trust in HCPs, reinforcing existing concerns about digital misinformation and its role in shaping patient expectations.^54^ These findings highlight the need for public health initiatives that facilitate safe communication in health consultations.

The social impact of cyber-victimisation was another key finding, with 22.41% of HCPs reporting withdrawal from online communities as a consequence, and provided further insight in the themes on how such social isolation could be online, offline, or impacts trust with healthcare providers. This aligns with existing studies that show how online abuse can lead to social isolation, particularly for those who rely on digital platforms for peer support.^55^ Given the importance of online support networks, addressing cyber-victimisation is critical in ensuring continued access to social support,^56,57^ which is a wider and a complex issue that requires multidisciplinary interventions.

The role of age in cyber-victimisation remains complex. Some respondents viewed older individuals as more vulnerable due to limited digital literacy, while others considered them less at risk due to lower social media engagement. This dual perception is consistent with issues in estimating the older age group’s digital literacy and skills.^58^ Additionally, the study identified that certain conditions, such as Long COVID, were disproportionately affected by cyber abuse. The victimisation of different long-term conditions is documented,^10^ however, targeting people with long-term COVID is a new finding which might need further research.

The study also revealed a notable contrast between participants. While some expressed uncertainty or disbelief about the relevance of cyber-victimisation to healthcare, others described direct encounters with affected patients and recognised clear health impacts. One particularly unexpected insight was the mention of cyber-victimisation surfacing in anaesthesia setting. This demonstrates the effect of mental health impact and suggests that patients may bring such concerns to a wider range of professionals than previously assumed. The diversity of these professionals, which included GPs, nurses, psychologists, and paramedics, was surprising given previous concerns about trust in support channels in multiple settings in the UK^16,31,53^ and the potential for stereotyping of people with disabilities and chronic conditions.^59^ This contrast points to an important tension in the field: while some professionals are attuned to the implications of cyber-victimisation, others may overlook or minimise its relevance, suggesting gaps in training and awareness.

A considerable proportion of HCPs (58.44%) believed that research-informed training on cyber-victimisation’s impact on their patients would be beneficial. The preference for interactive media (44.19%) and workshops (30.23%) suggests a demand for engaging, practical learning formats. However, some professionals stated that they had never encountered cyber-victimisation in their practice or perceived it as having minimal impact. This disparity highlights a potential gap in awareness and recognition of cyber-victimisation, echoing findings from a previous systematic review which identified inconsistencies in defining and estimating cyber-victimisation and called for facilitating professional communication and structured public health initiatives.^11^

HCPs identified key barriers to intervention, including lack of training, limited consultation time, and uncertainty about their role in addressing cyber-victimisation. This aligns with the broader challenges of limited consultation times and GPs’ workload in the UK.^60,61^ Thus, while HCPs are best suited to anticipate and manage health impacts, future interventions should ideally address the bigger picture for the health system, integrate digital harm awareness into public health strategies, and recognise the multidisciplinary nature of support needed.

While this study confirms the relevance of cyber-victimisation in clinical encounters, further guidance is needed to support healthcare professionals in recognising and responding to this issue. Rather than routine screening, a more feasible approach may involve selective enquiry based on clinical judgement, observable signs of psychological distress, or if patients raise concerns during consultations.^62^ When discussing these experiences, a non-judgemental and empathetic communication style is critical, particularly given the known risks of victim-blaming, stereotyping, and limited awareness among professionals.^63-66^

To respond to the identified training gap, future initiatives should focus on equipping healthcare professionals with core competencies such as recognising signs of cyber-victimisation, understanding its impact on physical and psychological health, communicating sensitively with affected patients, and identifying appropriate referral pathways. In terms of delivery, participants in this study expressed a preference for interactive materials such as short modules and workshops. Training could initially be piloted as a prototype co-developed with academic experts, patient advocates, and representatives from referral networks including NGOs and law enforcement.^31,53,67^ While it may be premature to tailor content to individual professional groups, elements such as referral protocols may require more emphasis for GPs.^61^ Given the complexity of the issue, a multidisciplinary approach should underpin the training to foster collaborative responses.

Referral pathways should be tailored to the specific context and type of harm disclosed. This may include referral to mental health services for psychological impacts, specialist medical services if the underlying chronic condition has worsened, safeguarding teams or law enforcement in cases involving abuse or threat, or voluntary sector organisations for advocacy and peer support. Due to the complexity of needs, a multidisciplinary approach is essential.^11^ Although cyber-victimisation may not always fall within routine clinical protocols, the role of healthcare professionals lies in preventing secondary health consequences by acting early and effectively. From this perspective, cyber-victimisation should be viewed as a public health concern and incorporated into broader psychosocial frameworks of care.^15,68,69^

While some HCPs have more frontline roles in supporting people who are targeted,^7^ additional statistical analyses found no significant associations between professional background and reported encounters with patients experiencing cyber-victimisation. However, these exploratory analyses suggest that awareness or exposure may not differ markedly across these categories, the numbers within subgroups were low to reach conclusions. Hence, further research is needed to examine what shapes professionals’ recognition of cyber-victimisation and disclosure by patients.

This study has acknowledged limitations. First, the sample could have been influenced by the recruitment methods, which may affect the generalisability of the findings.^70^ A formal power calculation was not conducted, because the purpose of the survey was to generate descriptive statistics rather than support inferential analysis. A mixed-methods approach was used to address this potential limitation. The qualitative themes added depth, context, and meaning to the quantitative results, and findings from both components were integrated in the discussion. One of the strengths of mixed-methods research is its ability to explore emerging or under-researched areas, with each methodology complementing and enhancing the other to provide a more comprehensive understanding.

Second, self-reported data may be influenced by recall bias or personal perceptions of cyber-victimisation,^31^ hence the integration of findings helped to provide a more comprehensive understanding. Finally, the study primarily relied on asynchronous survey responses, and further in-depth qualitative methods could provide deeper insights into experiences and training needs. However, the study is the first one to cover this issue in the UK, and the study design suited the targeted audience due to the ongoing issue of time constraints and workload impacting the health workforce in the UK.^60,61^

## Conclusion

This study provides novel evidence that healthcare professionals across a range of roles encounter patients with chronic conditions and/or disabilities who have experienced cyber-victimisation. While the nature and depth of these encounters vary, those with direct experience highlighted clear health and social impacts. The variability in awareness and perceived relevance among HCPs may reflect differences in clinical exposure, patient populations, or professional roles. These findings highlight how cyber-victimisation can compound existing health challenges, with potential consequences for emotional wellbeing, self-management, and trust in healthcare. As such, cyber-victimisation must be considered a public health issue requiring appropriate recognition and interventions. Importantly, the results support the need for targeted, research-informed training for healthcare professionals to recognise, understand, and respond to cyber-victimisation within routine care settings.
